# A PKA activity sensor for quantitative analysis of endogenous GPCR signaling *via* 2-photon FRET-FLIM imaging

**DOI:** 10.3389/fphar.2014.00056

**Published:** 2014-04-02

**Authors:** Yao Chen, Jessica L. Saulnier, Gary Yellen, Bernardo L. Sabatini

**Affiliations:** ^1^Howard Hughes Medical InstituteBoston, MA, USA; ^2^Department of Neurobiology, Harvard Medical SchoolBoston, MA, USA

**Keywords:** PKA, FLIM, neuromodulation, cAMP, FLIM-AKAR, GPCR, glutamate, dendritic spine

## Abstract

Neuromodulators have profound effects on behavior, but the dynamics of their intracellular effectors has remained unclear. Most neuromodulators exert their function *via* G-protein-coupled receptors (GPCRs). One major challenge for understanding neuromodulator action is the lack of dynamic readouts of the biochemical signals produced by GPCR activation. The adenylate cyclase/cyclic AMP/protein kinase A (PKA) module is a central component of such biochemical signaling. This module is regulated by several behaviorally important neuromodulator receptors. Furthermore, PKA activity is necessary for the induction of many forms of synaptic plasticity as well as for the formation of long-term memory. In order to monitor PKA activity in brain tissue, we have developed a 2-photon fluorescence lifetime imaging microscopy (2pFLIM) compatible PKA sensor termed FLIM-AKAR, which is based on the ratiometric FRET sensor AKAR3. FLIM-AKAR shows a large dynamic range and little pH sensitivity. In addition, it is a rapidly diffusible cytoplasmic protein that specifically reports net PKA activity *in situ*. FLIM-AKAR expresses robustly in various brain regions with multiple transfection methods, can be targeted to genetically identified cell types, and responds to activation of both endogenous GPCRs and spatial-temporally specific delivery of glutamate. Initial experiments reveal differential regulation of PKA activity across subcellular compartments in response to neuromodulator inputs. Therefore, the reporter FLIM-AKAR, coupled with 2pFLIM, enables the study of PKA activity in response to neuromodulator inputs in genetically identified neurons in the brain, and sheds light on the intracellular dynamics of endogenous GPCR activation.

## Introduction

Neuromodulators such as dopamine, serotonin and opioids have profound effects on neurons, circuits and behavior (Hikosaka et al., 2008; Kreitzer and Malenka, 2008; Le Merrer et al., 2009). Perturbations in neuromodulator function have been linked to diseases such as Parkinson's, and neuromodulator therapy has been used to treat diseases such as depression and schizophrenia (Albin et al., 1989; Nemeroff and Owens, 2002; Le Merrer et al., 2009).

Extensive biochemical characterization has identified protein kinase A (PKA) as a convergent site of action for many neuromodulators and neurotransmitters (Greengard, 2001). Most neuromodulators exert their function *via* G-protein-coupled receptor (GPCRs); neurotransmitters including glutamate and GABA can also act *via* metabotropic receptors that are GPCRs. GPCRs coupled to Gαs and Gαi produce up- and down-regulation of adenylate cyclase (AC) activity, respectively. Activated AC produces cAMP whose accumulation activates PKA. Thus, Gαs- and Gαi-coupled GPCRs bidirectionally change PKA activity (Greengard, 2001). PKA, in turn, modulates synaptic transmission, long-term plasticity, learning and memory, and has been implicated in a number of neurodegenerative and psychiatric diseases (Brunelli et al., 1976; Kandel and Abel, 1995; Davis, 1996; Brandon et al., 1997; Tzounopoulos et al., 1998; Shaywitz and Greenberg, 1999; Baxter, 2003; Skeberdis et al., 2006; Tronson et al., 2006; Shen et al., 2008; Zhong et al., 2009; Higley and Sabatini, 2010). Therefore, PKA can act as a potential integrator of diverse cellular inputs to mediate synaptic and cellular changes.

**Figure 1:**
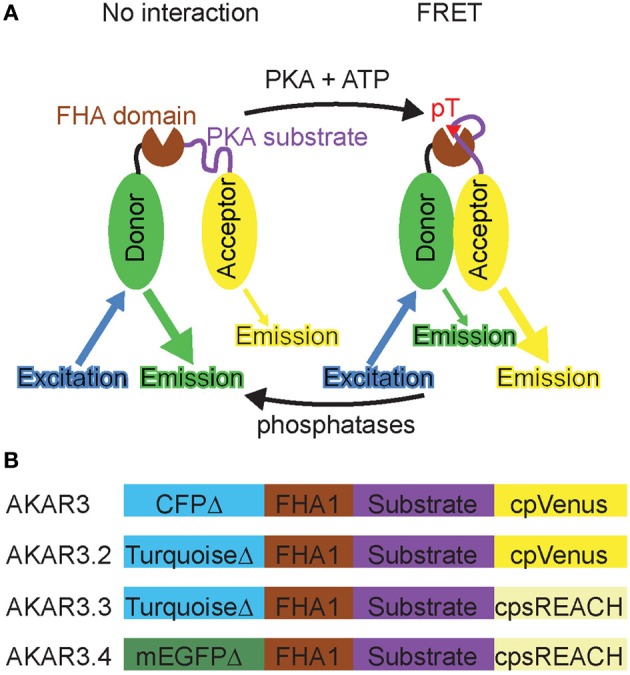
**Development of a PKA sensor compatible with 2-photon Fluorescence Lifetime Imaging Microscopy (2pFLIM). (A)** Diagram illustrating how PKA activity induces FRET in the reporter. Upon phosophrylation by PKA, the substrate region binds FHA domain, bringing the donor and acceptor together and resulting in FRET. The phosphorylated reporter leads to an increase of acceptor:donor emission ratio, as well as a decrease in donor fluorescence lifetime because of an additional energy transfer pathway. **(B)** Schematic of the original PKA reporter AKAR3 (Allen and Zhang, 2006) and three new PKA reporters.

The neurotransmitter and neuromodulator inputs that activate PKA carry important timing information—for example, dopamine release in the striatum is thought to modulate glutamatergic synapses that are active near the time of release and hence reinforce recently executed behaviors (Schultz, 1998; Berke and Hyman, 2000). In addition, the activity of PKA in different subcellular compartments, such as dendritic spines, the cytoplasm, and the nucleus, phosphorylates different substrates and triggers different cellular responses. Therefore, in order to understand how PKA dynamically integrates ongoing inputs to affect cellular and synaptic function, it is necessary to measure both the timing and subcellular location of PKA activity in response to endogenous GPCR activation. A Förster Resonance Energy Transfer (FRET)-based PKA activity reporter, AKAR3, was developed for ratiometric imaging (Allen and Zhang, 2006). AKAR3 consists of a fusion of a donor fluorophore (truncated CFP), a phosphopeptide binding domain (FHA1), a consensus region of PKA substrates, and an acceptor fluorophore (circularly permuted Venus) (Figure 1A). When PKA is inactive, the donor and acceptor fluorophores are far apart, resulting in low FRET. Upon phosphorylation by PKA, the substrate region binds the phosphopeptide binding domain FHA1, bringing the donor and acceptor fluorophores together and resulting in high FRET. Conversely, dephosphorylation by phosphatases reverses the process. Thus, AKAR3 serves as a PKA substrate to report the balance between PKA and phosphatases, which we here refer to as net PKA activity.

Despite the success of AKAR3 and its derivatives as a ratiometric FRET reporter of PKA activity (Allen and Zhang, 2006; Vincent et al., 2008; Depry et al., 2011; Lam et al., 2012), it poses challenges for quantifying FRET in brain tissue, notably the difficulty to use AKAR3 with two photon (2p) microscopy. An alternative to ratiometric imaging for FRET measurement is Fluorescence Lifetime Imaging Microscopy (FLIM). FLIM only measures the donor, and not acceptor fluorescence, and the fluorescence lifetime of the donor reflects the FRET interaction between the donor and acceptor: increased FRET from donor to acceptor is directly reflected as a reduced fluorescence lifetime of the donor (Supplementary Figure 1). A FLIM reporter can potentially alleviate the challenge of 2p ratiometric imaging such as spectral bleedthrough and wavelength-dependent scattering, and allows us to monitor the spatiotemporal dynamics of net PKA activity in brain tissue.

Here, we report the development of a 2pFLIM compatible sensor FLIM-AKAR that reports the balance of PKA and phosphatase activity. The new reporter shows a large dynamic range, little pH sensitivity, and is specific for PKA. In addition, it acts as a rapidly diffusible cytoplasmic protein. The reporter can be introduced into neurons *via* biolistic transfection, *in utero* electroporation, or viral infection, and can report net PKA activity in subcellular compartments including the nucleus and dendritic spines. Furthermore, we engineered a Cre recombinase (Cre)-dependent FLIM-AKAR plasmid which can be packaged into adeno–associated viruses (AAV), allowing expression in genetically identified neurons. FLIM-AKAR signals robustly in response to AC activation, endogenous GPCR activation and induction of long-term potentiation at individual dendritic spines, and it shows differential kinetics of PKA signaling across subcellular compartments. Therefore, FLIM-AKAR, combined with 2pFLIM, provides an essential tool to quantitatively monitor the intracellular dynamics of signaling of a large class of GPCRs with high spatial temporal resolution.

## Results

### Generation and comparison of constructs for 2pFLIM imaging of net PKA activity

Although AKAR3 and its derivatives were successfully used for ratiometric imaging of PKA activity (Allen and Zhang, 2006; Vincent et al., 2008; Depry et al., 2011; Lam et al., 2012), they are not suitable for imaging in brain tissue with 2pFLIM due to spectral bleedthrough and the properties of the donor fluorophore. Therefore, we changed the donor-acceptor pair of AKAR3 in order to make a 2pFLIM reporter of PKA activity with the following characteristics: (1) brighter donor fluorescence to improve the signal-to-noise ratio; (2) darker acceptor fluorescence to minimize contamination into the donor channel; (3) donor fluorophore with less lifetime rundown; and (4) free donor fluorophore with lifetime distribution well fit by a single exponential, which makes curve fitting easy so that we can calculate FRET to free donor ratio (Supplementary Figure 1). The considerations for choosing the donor-acceptor pair are detailed in Supplementary Table 1.

**Figure 2:**
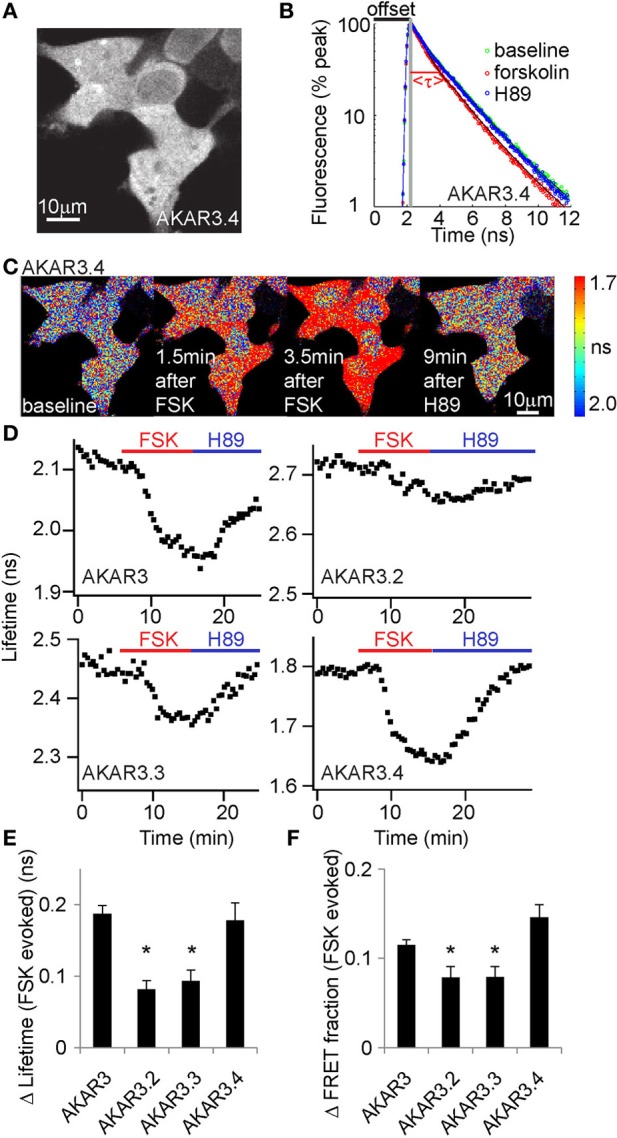
**AKAR3.4 reports PKA activity with the largest dynamic range in 2pFLIM measurements. (A)** Image showing AKAR3.4 expression in Human Embryonic Kidney (HEK) cells 1 day after transfection. **(B)** Fluorescence decay curves following pulsed excitation. Application of the adenylate cyclase activator forskolin (50 μM) in AKAR3.4-transfected HEK cells results in faster decay and a decrease in lifetime, whereas application of the PKA inhibitor H89 (10 μM) reverses the decay curve to baseline. The offset arrival time, the time from the detection of photon excitation from the laser to the detection of the same pulse from the photomultiplier tube, is labeled in black, and the actual mean fluorescent lifetime τ is labeled in red. **(C)** Lifetime heat map of HEK cells transfected with AKAR3.4, showing lifetime changes induced by forskolin (FSK, 50 μM) followed by H89 (10 μM) treatment. **(D)** Example plots showing lifetime responses of the four different reporters to forskolin (50 μM, red bar) followed by H89 (10 μM, blue bar) treatment. Experiments were done in HEK cells transfected with the respective reporters. **(E)** Amplitudes of Δlifetime between baseline and forskolin (50 μM) treatments. **(F)** Change in the FRET fraction of photons (P_FRET_ based on the annotation in Supplementary Figure 1) between baseline and forskolin (50 μM) treatments. For **(E,F)**, *n* = 12, 10, 14, and 12 cells for AKAR, AKAR3.2, AKAR3.3, and AKAR3.4 respectively. ^*^p < 0.003 when the amplitudes of the reporter and AKAR3.4 were compared (α = 0.017 for a familywise error rate of 0.05).

We made three constructs to meet the above criteria (Figure 1B) and determined empirically which gave the best dynamic range. All of the constructs showed good expression in HEK293T cells (Figure 2A and data not shown). Following addition of the AC activator forskolin to drive PKA activity, all of the constructs showed decreased fluorescence lifetimes and increased FRET fractions of photons, consistent with higher FRET upon reporter phosophorylation (Figures 2B–D). Subsequent application of the PKA inhibitor H89 reversed these changes. Of the three new reporters, AKAR3.4 showed the largest amplitude change upon AC activation, as measured by both lifetime changes (Δlifetime) (Figure 2E) and changes in the FRET fractions of photons (Figure 2F).

**Figure 3:**
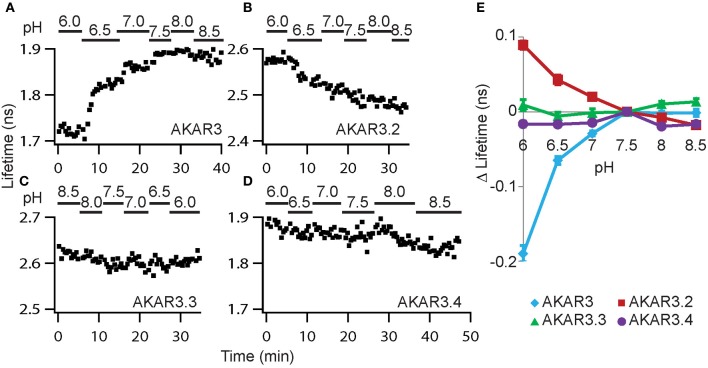
**AKAR3.4 shows little sensitivity to pH. (A–D)** Example plots showing responses of the four reporters to pH changes. Experiments were performed in HEK cells at room temperature in the presence of nigericin (5 μM) and the PKA inhibitor H89 (10 μM). Extracellular solutions buffered to different pH values were applied. **(E)** Summary graph showing Δlifetime in response to different pH in HEK cells. Δlifetime was measured relative to that at pH 7.5. *n* = 8, 8, 7, and 7 cells for AKAR3, AKAR3.2, AKAR3.3, and AKAR3.4 respectively. Graphs show mean and SEMs.

Since intracellular pH can respond to electrical and biochemical signals in neurons (Berg et al., 2009; Tantama et al., 2011; Raimondo et al., 2012; Rathje et al., 2013) and can also affect protein fluorescence, we characterized how the different constructs responded to changes in pH. To manipulate intracellular pH, the K^+^/H^+^ ionophore nigericin was used to permeabilize the plasma membrane to protons (Thomas et al., 1979), and extracellular solutions buffered to different pH values were applied. In addition, the cell-permeable PKA inhibitor H89 was included to eliminate any potential pH-induced change in PKA activity. Of the four constructs, AKAR3 and AKAR3.2 showed large changes with varying pH, whereas AKAR3.3 and AKAR3.4 showed little pH sensitivity (Figure 3). AKAR3.4 contains truncated monomeric eGFP (meGFPΔ) as the donor and circularly permuted dark YFP (cpsREACH) as the acceptor. Therefore, in addition to the largest amplitude of Δlifetime in response to AC activation and little pH sensitivity, AKAR3.4 also fulfilled the criteria outlined above, showing brighter donor fluorescence (Piston et al.; Shaner et al., 2007), darker acceptor fluorescence (Ganesan et al., 2006; Murakoshi et al., 2008), and less donor lifetime rundown than the original AKAR3 (Figure 2D). meGFP also shows a single exponential decay (Murakoshi et al., 2008), whereas CFP in AKAR3 shows multi-exponential decay. Thus, AKAR3.4 is the most suitable PKA activity reporter for quantitative 2pFLIM imaging. We termed it FLIM-AKAR and used it for the remainder of the study.

### Diffusion and specificity of FLIM-AKAR

**Figure 4:**
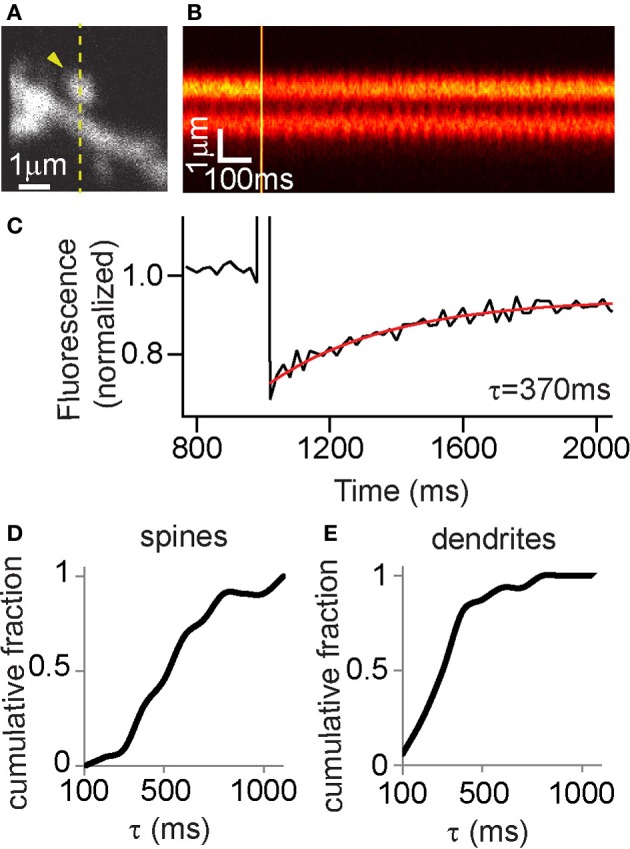
**FLIM-AKAR shows similar diffusion properties to a rapidly diffusing cytoplasmic protein. (A–E)** Fluorescence Recovery After Photobleaching (FRAP) experiments to measure the spreading of FLIM-AKAR. **(A)** Image of FLIM-AKAR donor fluorescence of a dendritic region of a hippocampal CA1 neuron cultured in organotypic slices. The arrowhead shows the spine that was photobleached. The dashed line shows the region being imaged by line scan. **(B)** Fluorescence measured in line scans for the region indicated by dashed line in **(A)**. **(C)** Quantification of FRAP for the spine shown in **(A,B)**. The trace was an average of 4 acquisitions. The red trace shows curve fitting with a single exponential decay. **(D,E)** Cumulative distribution of τ from FRAP experiments to examine FLIM-AKAR spreading from spines **(D)** and for aspiny regions of dendrites **(E)** in hippocampal CA1 neurons.

FLIM-AKAR is a substrate for PKA phosphorylation and is not targeted to any subcellular compartments. Therefore, in order to understand if spatial patterns of the FLIM-AKAR response represent subcellular spatial differences in net PKA activity in real time, it is necessary to characterize its diffusional properties. To do so, we performed fluorescence recovery after photobleaching (FRAP) in CA1 pyramidal neurons to measure the replenishment of FLIM-AKAR after bleaching (Figures 4A–C). FRAP in aspiny regions of dendrites revealed a time constant of recovery of 323 ± 42 ms (mean ± standard error of the mean (SEM), *n* = 16 regions of dendrites, Figure 4E). FRAP in the heads of mushroom-like spines revealed time constants of recovery of 544 ± 49 ms (mean ± SEM, *n* = 22 spines, Figure 4D). This is comparable to time constants of diffusional equilibration across spine head for other diffusible proteins of similar size (Pologruto et al., 2004; Bloodgood and Sabatini, 2005; Harvey et al., 2008; Lee et al., 2009; Yasuda and Murakoshi, 2011). Therefore, we conclude that FLIM-AKAR behaves as a rapidly diffusible cytoplasmic protein. Thus, if a more persistent lifetime change is observed in the spine in a given experiment compared with the diffusion time constant, it is attributable to active PKA or phosphatase activity in the spine (see below).

**Figure 5:**
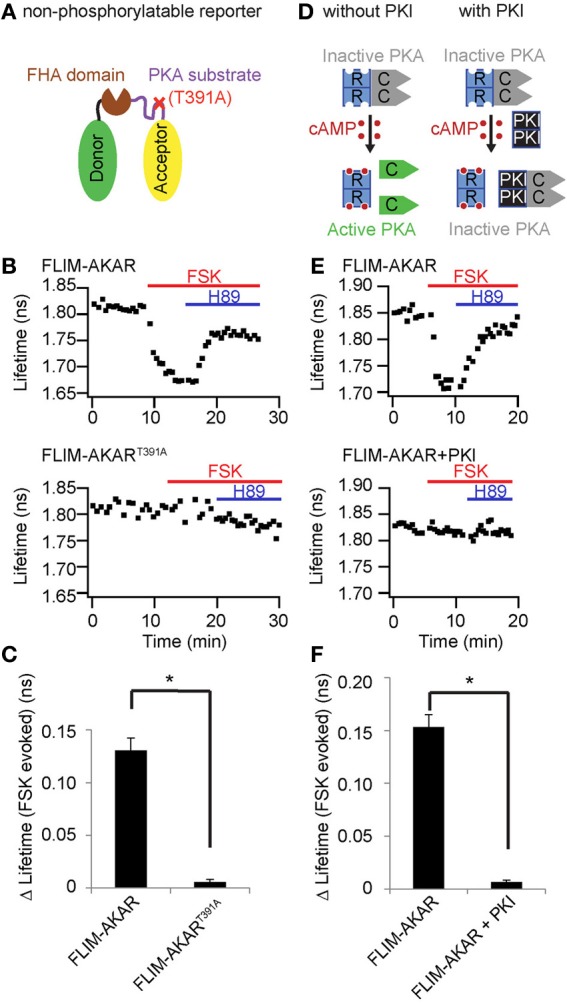
**FLIM-AKAR is specific for PKA following adenylate cyclase activation by forskolin**. All experiments were performed in HEK cells. Red line indicates bath application of 50 μM forsklin (FSK) and blue line indicates bath application of 10 μM H89. **(A)** Schematic illustrating introduction of the point mutation T391A that renders the PKA phosophorylation site of FLIM-AKAR non-functional. **(B)** Example plots showing FLIM-AKAR lifetime response to adenylate cyclase activation by forskolin (red) and subsequent PKA inhibition by H89 (blue) (top), and the lack of a response from the non-phosphorylatable point mutant FLIM-AKAR^T391A^ (bottom). **(C)** Summary bar graph showing Δlifetime from baseline to forskolin treatment for FLIM-AKAR (*n* = 9 cells) and the non-phosphorylatable mutant of FLIM-AKAR (*n* = 11 cells). ^*^p < 10^−5^. **(D)** Schematic illustrating how PKI inhibits PKA activity. Without PKI, binding of cAMP to the regulatory subunits of PKA (R) frees the catalytic subunits (C), resulting in activation of PKA. With PKI, even though cAMP binding to the regulatory subunits dissociates them from the catalytic subunits, PKI can bind to the catalytic subunits of PKA and inhibit their activity. **(E)** Example plot showing FLIM-AKAR response to adenylate cyclase activation and subsequent PKA inhibition (top), and the lack of a lifetime response by FLIM-AKAR when it was co-transfected with PKIα (bottom). **(F)** Summary bar graph showing Δlifetimes from baseline to forskolin treatment for FLIM-AKAR, in the absence (*n* = 18 cells) and presence (*n* = 10 cells) of PKI. ^*^p < 10^−9^.

Previous versions of AKAR do not respond to CamKII or PKC. In addition, in the presence of the PKA inhibitor H89 or the PKA inhibitor peptide (PKI), they do not respond to isoproterenol activation of the Gαs-coupled β-adrenergic receptors (Zhang et al., 2001, 2005; Allen and Zhang, 2006), confirming their specificity for PKA. We also tested the specificity of FLIM-AKAR to report changes in net PKA activity. First, we introduced a point mutation at the phosphorylation site in the substrate region. This mutant reporter (FLIM-AKAR^T391A^) did not respond to forskolin or H89 application (Figures 5A–C), indicating that this phosphorylatable residue is required for FLIM-AKAR response to AC activation. Second, FLIM-AKAR did not respond to activation of PKC by phorbol 12, 13-dibutyrate (PDBu), confirming that the reporter is not sensitive to PKC activation (*n* = 9 cells). Third, addition of H89 to inhibit PKA largely reversed forskolin-induced Δlifetime (Figures 5B,E). Finally, in the presence of the PKA inhibitor peptide PKI (Ashby and Walsh, 1972, 1973; Dalton and Dewey, 2006), FLIM-AKAR did not respond to addition of forskolin or H89 (Figures 5D–F). Taken together, these results demonstrate that FLIM-AKAR does not respond to PKC, and is specific for PKA following AC activation by forskolin.

### *In vivo* expression and response to endogenous receptor activation

**Figure 6:**
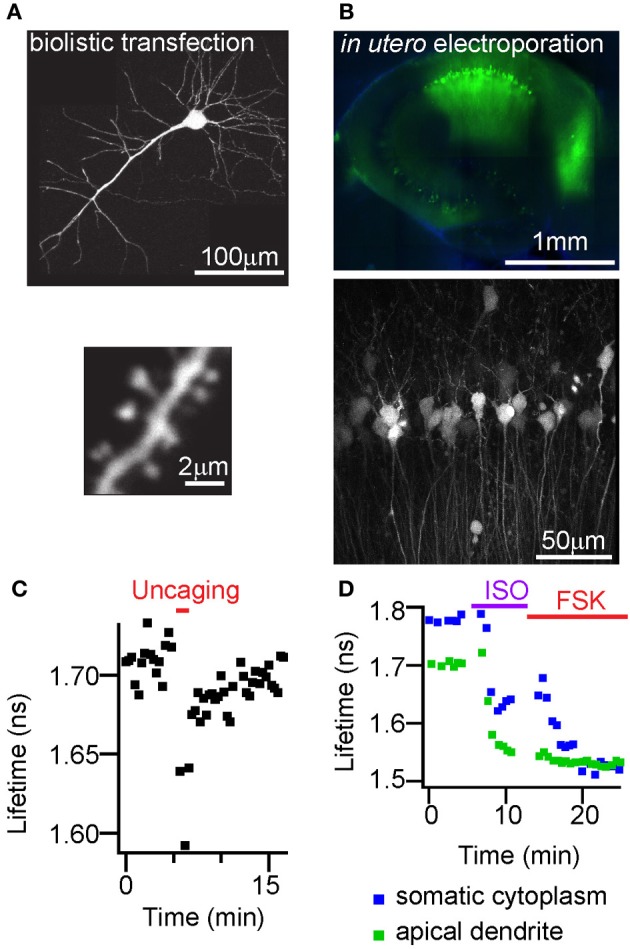
**FLIM-AKAR shows high expression with different transfection methods and lifetime responses to glutamate or GPCR activation in the hippocampus. (A)** Images showing a hippocampal CA1 neuron (top), dendrite and spines (bottom) from an organotypic slice transfected with FLIM-AKAR with biolistic method. **(B)** Image showing a 300 μm acute hippocampal slice expressing FLIM-AKAR in CA1 region after *in utero* electroporation. **(C)** Example plot of lifetime change of FLIM-AKAR in a stimulated spine in response to photolysis of caged glutamate adjacent to the spine. A CA1 pyramidal neuron in an organotypic hippocampal slice was biolistically transfected with FLIM-AKAR, and the stimulated spine shows enlargement following 2-photon photolysis of caged glutamate (MNI-glutamate). The temporal window of uncaging is indicated in the red bar above. **(D)** Lifetime response of FLIM-AKAR upon isoproterenol (1 μM, ISO) treatment to activate β-adrenergic receptors followed by forskolin (50 μM, FSK) treatment to activate adenylate cyclases. The experiment was done in acute hippocampal slice expressing FLIM-AKAR in CA1 region after *in utero* electroporation.

**Figure 7:**
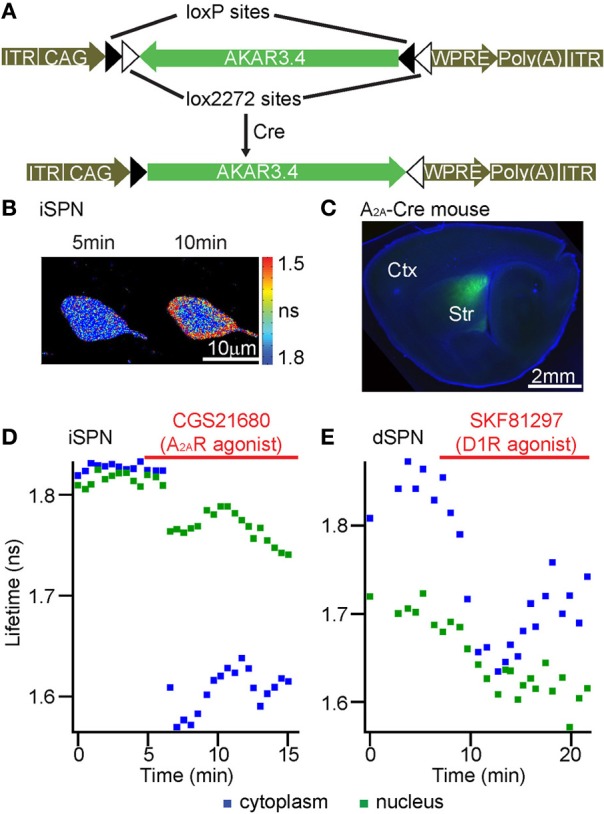
**FLIM-AKAR can be targeted to genetically defined cell types and reports modulation of PKA by endogenous GPCRs in the striatum. (A)** AAV plasmid map showing how Cre recombinase leads to AKAR3.4 expression. **(B–D)** A Cre-dependent AAV virus carrying FLIM-AKAR was delivered to the striatum of an *Adora2a* BAC-Cre mouse. **(B)** Lifetime heat map of an indirect pathway striatal spiny projection neuron (iSPN) in an acute striatal slice. Following A_2A_R activation by its agonist CGS21680, cytoplasmic FLIM-AKAR became rapidly phosphorylated, while nuclear FLIM-AKAR showed a slower response. **(C)** A parasagittal brain section showing FLIM-AKAR expression in the striatum. **(D)** Example plots showing modulation of PKA by A_2A_R in an iSPN. 1 μM CGS21680 was used to activate A_2A_R. **(E)** A Cre-dependent AAV virus carrying FLIM-AKAR was delivered to the striatum of a *Drd1* BAC-Cre mouse to target direct pathway SPNs (dSPNs). Example plots showing modulation of PKA by SKF81297 in a dSPN. 1 μM SKF81297 was used to activate D1R.

We tested if FLIM-AKAR shows sufficiently high expression *in vivo* and adequate characteristics to report changes in PKA activity following glutamate or endogenous neuromodulator receptor activation in thick brain tissue. We introduced FLIM-AKAR with three different methods into brain tissue. First, we transfected FLIM-AKAR biolistically in organotypic slices (Figure 6A). Second, we introduced FLIM-AKAR by *in utero* electroporation into the cortex (data not shown) or hippocampus (Figure 6B). Third, we generated an adeno-associated virus (AAV) that expresses FLIM-AKAR in a Cre-recombinase (Cre) dependent manner, and injected the virus into mice expressing Cre in specific cell populations (Figures 7A–C). In all three cases, FLIM-AKAR showed robust expression, and can be seen in the soma and dendrites, including dendritic spines. Thus, FLIM-AKAR is a versatile reporter that can be introduced by a variety of methods to achieve expression *in vivo*.

In order to test if FLIM-AKAR has the sensitivity to respond to spatiotemporally precise and physiologically relevant stimuli, we delivered glutamate by 2-photon photolysis of photoactivatable glutamate (MNI-glutamate) adjacent to individual dendritic spines. The stimulated spine enlarged in response to a structural plasticity protocol previously reported in multiple studies to induce potentiation of the associated postsynaptic terminal (Matsuzaki et al., 2004; Steiner et al., 2008; Murakoshi et al., 2011). Strikingly, this resulted in a decrease in fluorescence lifetime during and after photoactivation to release glutamate (Figure 6C). Thus, 2-photon photolysis of photoactivatable glutamate leads to a net increase of PKA activity in the stimulated spine, and FLIM-AKAR can report net PKA activity in small volumes such as the spine in response to spatiotemporally precise stimuli.

To test if FLM-AKAR can respond to endogenous GPCR activation, we used isoproterenol to activate the Gαs-coupled β-adrenergic receptors in hippocampal CA1 pyramidal neurons (Figure 6D). Fluorescence lifetime decreased in both soma and dendrites, indicating an increase in net PKA activity. Subsequent application of forskolin resulted in a further lifetime decrease in somatic cytoplasm but not apical dendrites, indicating that isoproterenol induced maximal reporter phosphorylation in apical dendrites and partial AC activation in somatic cytoplasm.

Finally, we targeted FLIM-AKAR to genetically defined cell types and examined FLIM-AKAR response to endogenous GPCR activation in distinct cell types. For this purpose, we tested FLIM-AKAR in the striatum, a subcortical brain region that receives a large number of neuromodulator inputs (Kreitzer and Malenka, 2008; Lerner and Kreitzer, 2011). In the striatum, different neuromodulator receptors are preferentially expressed in different types of striatal spiny projection neurons (SPNs). Indirect pathway SPNs (iSPNs) preferentially express the Gαs-coupled adenosine receptor A_2A_R, whereas direct pathway SPNs (dSPNs) preferentially express the Gαs-coupled dopamine D1 receptors (D1R) (Kreitzer and Malenka, 2008; Lerner and Kreitzer, 2011). Thus, we would expect that activation of the preferentially expressed Gαs-coupled receptors would result in net PKA activity in the corresponding type of SPNs. Here, we first injected AAV encoding Cre-dependent FLIM-AKAR into the striatum of *Adora2a* BAC-Cre mice (Heintz, 2004; Durieux et al., 2009) to target iSPNs. FLIM-AKAR showed robust expression in the striatum (Figures 7B,C). Application of the A_2A_R agonist CGS21680 decreased lifetime in both the cytoplasm and the nucleus (Figure 7D). We also used *Drd1* BAC-Cre mice (Heintz, 2004; Gong et al., 2007), and injected virus carrying the Cre-dependent FLIM-AKAR into the striatum in order to target dSPNs. The D1R agonist SKF81297 produced a rapid lifetime decrease in the cytoplasm and a slower decrease in the nucleus (Figure 7E). Therefore, FLIM-AKAR can be targeted to genetically defined cell types, was able to report regulation of PKA activity by distinct endogenous GPCR in these cell types, and revealed differential kinetics in different subcellular compartments.

## Discussion

In order to image the spatiotemporal dynamics of net PKA activity in brain tissue, we developed and characterized a 2pFLIM-compatible PKA reporter FLIM-AKAR for quantitative imaging. FLIM-AKAR shows a large dynamic range of FLIM signals and little pH sensitivity. In addition, it is a diffusible cytoplasmic protein and shows specificity to PKA following forskolin stimulation. Finally, it can be targeted to genetically defined neurons, and can report net PKA activity in response to both glutamate and endogenous neuromodulator GPCR activation. Thus, FLIM-AKAR enables the study of PKA activity in response to neurotransmitter and neuromodulator inputs in genetically identified cell types, and promises to shed light on the intracellular dynamics of endogenous GPCR signaling.

### A PKA reporter for 2pFLIM

2p microscopy facilitates fluorescence imaging within living tissue such as the brain due to its diminished sensitivity to light scattering and the restriction of fluorophore excitation to the focus. However, it poses considerable challenges for ratiometric FRET imaging. This is mainly because the 2p excitation spectra of many fluorophores are broad, resulting in overlapping donor and acceptor excitation and emission spectra, which leads to noisy intensity measurements since the noise is amplified during correction for spectral bleedthrough during ratiometric imaging (Yasuda et al., 2006).

2pFLIM is advantageous over ratiometric imaging for FRET measurement in thick brain tissue (Bastiaens and Squire, 1999; Yasuda, 2006). Since 2pFLIM directly measures the lifetime of the donor fluorophore, it is insensitive to many of the technical challenges that accompany ratiometric FRET imaging in brain tissue such as fluorophore concentration and wavelength-dependent light scattering. 2pFLIM is also not sensitive to artifacts introduced by low intensity measurements in ratiometric FRET, notably focus change, tissue movement, and differential photobleaching. Furthermore, with the use of dark acceptor fluorophores (Ganesan et al., 2006; Murakoshi et al., 2008), 2pFLIM avoids the propagation of noise due to corrections for spectral bleedthrough and ectopic excitation of fluorophores involved in ratiometric FRET. Finally, 2pFLIM is inherently quantitative: donor lifetime analysis directly gives the fractions of fluorophores that are free and that have undergone FRET. Thus, although the PKA reporter AKAR3 and two of its derivatives have proven valuable for ratiometric FRET imaging (Allen and Zhang, 2006; Depry et al., 2011; Lam et al., 2012), a 2pFLIM reporter of PKA activity offers advantages for the study of endogenous GPCR signaling in intact brain tissue.

The 2pFLIM compatible reporter FLIM-AKAR shows robust responses to neuromodulator and neurotransmitter receptor activation in brain tissue. We found net PKA activity increase with activation of β-adrenergic receptors in hippocampal CA1 neurons, of A_2A_R in iSPNs, and of D1R in dSPNs, all of which are consistent with the Gαs-coupling of these receptors (Lefkowitz, 2007; Lerner and Kreitzer, 2011). Beside neuromodulator receptor activation, FLIM-AKAR also revealed an increase in net PKA activity in response to glutamate, potentially due to metabotropic glutamate receptor activation (Wang and Zhuo, 2012). Thus, based on the characterization of the reporter and its demonstrated utility in brain tissue, FLIM-AKAR is suitable for studying how PKA activity responds to endogenous GPCR signaling in the brain.

For future use of FLIM-AKAR, a couple of points should be noted. First, we confirmed the specificity of FLIM-AKAR for PKA with multiple methods (Figure 5). However, true specificity of any reporter has to be demonstrated for each biological application and experimental context, since each stimulus may elicit a different range of intracellular signals. Therefore, similar specificity tests should be performed for every new stimulus used in the future. Second, the expression level of a FLIM sensor is important. Since autofluorescence with a non-uniform lifetime distribution can contaminate actual signals, sufficient expression level is required for an accurate measurement of the lifetime of a sensor. The amount of expression required can be estimated by simulation of data combined with autofluorescence measurements in the biological system. In the biological experiments described here, the expression level is sufficient with all three methods of transfection.

### Diffusion of the 2pFLIM reporter and compartmentalized net PKA activity

Compartmentalization of intracellular signals is a key feature of neuronal processing that gives rise to synaptic specificity, and allows spatially segregated responses to different types of signals (Chen and Sabatini, 2012). Differential PKA kinetics has been demonstrated between cellular membrane, cytoplasm and nucleus (Dipilato et al., 2004; Allen and Zhang, 2006; Gervasi et al., 2007). Since FLIM-AKAR is a PKA substrate that is not specifically tagged, the diffusibility of the 2pFLIM reporter is important for interpreting the localization of PKA activity. Using FRAP we determined that FLIM-AKAR spreads like a diffusible cytoplasmic protein (Figure 4), with a time constant of hundreds of milliseconds.

Comparing time constants between FLIM-AKAR diffusion and biological experiments can reveal true temporal persistence and spatial compartmentalization of net PKA activity. Following structural plasticity induction, the lifetime decrease must be attributable to long-lasting net PKA activity within the spine, since the time of recovery to baseline lifetime (minutes) is far longer than the time constant of FLIM-AKAR diffusion from the spine (hundreds of milliseconds). In addition to the temporal persistence, we also demonstrate spatial heterogeneity in net PKA activity, both during basal states, and in the kinetics and amplitudes of response to GPCR activation (Figures 6, 7). These spatial differences are due to differential net PKA activity between dendrites, nucleus and somatic cytoplasm, since the differences persist on the order of minutes. This raises the interesting possibility that different physiological signals (different types, duration, and amplitude of stimulus) can change net PKA activity in different subcellular compartments, resulting in distinct functional consequences. FLIM-AKAR allows future investigations addressing the types of stimuli that result in net PKA activity changes in specific compartments, and the active mechanisms that maintain net PKA activity differences across subcellular compartments.

### Quantification of FLIM changes and relationship to GPCR signaling

An important advantage of FLIM is that it is inherently quantitative, such that the fitting of the fluorescence decay curve with a double-exponential decay function gives the fractions of donor fluorophores that are free and that have undergone FRET (for simplicity of notation, the convolution term with instrument response curve is not included here) (Yasuda, 2006):

$$F\left(t\right)=F_{0}(P_{free}{\text{e}}^{-\frac{t}{\tau_{free}}}+P_{FRET}{\text{e}}^{-\frac{t}{\tau_{FRET}}})$$

where *F*(*t*) is the fluorescence over time, *F*_0_ is the peak fluorescence, τ_*free*_ and τ_*FRET*_ are fluorescence lifetimes of donors that are free and that have undergone FRET respectively, and *P*_*free*_ and *P*_*FRET*_ are the corresponding fractions of these two species. In the case of the PKA reporter AKAR,

$$\overset{k_{PKA}}{\underset{k_{phosphatase}}{\left[AKAR]⇌[pAKAR\right]}}$$

where pAKAR stands for phosphorylated AKAR. If the expression level of FLIM-AKAR is low, steady state for the reporter can be achieved at all times. If the expression level is high, steady state may only be reached when the lifetime reaches a constant value, and the time course of the reporter lifetime between these constant values may lag the actual kinase or phosphatase activity. At steady state,

$$k_{PKA}\left[AKAR\right]=k_{phosphatase}\left[pAKAR\right]$$

Rearrangement of the equation gives the following:

$$\frac{\left[AKAR\right]}{\left[pAKAR\right]}=\frac{k_{phosphatase}}{k_{PKA}}$$

Therefore, knowing the fraction of pAKAR $\left(f_{pAKAR}=\frac{\left[pAKAR\right]}{\left[AKAR\right]+[pAKAR]}\right)$ would allow for the calculation of $\frac{k_{phosphatase}}{k_{PKA}}$. *f*_*pAKAR*_ can be interpolated from the fraction of free donors in a measurement. If *P*_*free*_(*experiment*) is the fraction of free donors in a given experiment, *P*_*free*_(*AKAR*) is the fraction of free donors measured when the reporters are not phosphorylated (for example, with the PKA inhibitor H89 or with the non-phosphorylatable mutant of FLIM-AKAR), *P*_*free*_(*pAKAR*) is the fraction of donors measured when the reporters are completely phosphorylated (for example, with forskolin, phosphodiesterase inhibitor and phosphatase inhibitor), then

$$f_{pAKAR}=\frac{P_{free}\left(AKAR\right)-P_{free}\left(experiment\right)}{P_{free}\left(AKAR\right)-P_{free}\left(pAKAR\right)}$$

Thus, the fraction of free donors from a given FLIM measurement can give the fraction of pAKAR, which gives the ratio of $\frac{k_{phosphatase}}{k_{PKA}}$ at steady state. Therefore, 2pFLIM measurement with FLIM-AKAR gives valuable quantitative information about the kinetic balance between PKA and phosphatase activity.

Taken together, our 2pFLIM compatible reporter FLIM-AKAR allows analysis of endogenous GPCR signaling in brain tissue, and can reveal previously unavailable quantitative information on the kinetic balance between phosphorylation and dephosphorylation of PKA substrates. Biologically, the reporter has revealed compartmentalization of net PKA activity in the dendrite, somatic cytoplasm and nucleus. With the multiple advantages of 2pFLIM imaging, FLIM-AKAR promises to reveal net PKA activity in response to neuromodulator inputs with high spatial temporal specificity.

## Materials and methods

### DNA constructs

The original pcDNA3-AKAR3 construct was a gift from Jin Zhang (Johns Hopkins University) (Allen and Zhang, 2006). AAV-AKAR3 was constructed by subcloning the coding region of pcDNA3-AKAR3 into the AAV vector AAV-ChR2-mCherry via EcoRI and BamHI sites. AAV-AKAR3 was used in imaging experiments in this study and referred to as AKAR3. AKAR3.2 was constructed by gene synthesis of codon-optimized truncated mTurquoise (Goedhart et al., 2010) (amino acid 1-227) together with part of the linker region between the donor and acceptor fluorophores, and subcloning of the synthesized fragment into AKAR3 via BamHI and PpumI (Genscript). AKAR3.3 was constructed by gene synthesis of part of the linker region between the donor and acceptor fluorophores together with circularly permuted sReaCh (Murakoshi et al., 2008) (^175^sReaCh^173^), and subcloning of the synthesized fragment into AKAR3.2 via SgrAI and EcoRI (Genscript). AKAR3.4 (also called FLIM-AKAR) was constructed by PCR amplification of truncated meGFP (amino acid 1-227) from the template GFP-sReaCh (Murakoshi et al., 2008) (Addgene) followed by recombination-based cloning with CloneEZ into AKAR3.3 to replace truncated mTurquoise (Genscript).

For the construction of the Cre-dependent reporter AAV-FLEX-FLIM-AKAR, the coding region of FLIM-AKAR was amplified by PCR and subcloned into AAV-FLEX-Arch-GFP (Atasoy et al., 2008; Chow et al., 2010) (Addgene Plasmid 22222) to replace Arch-GFP by recombination-based cloning with CloneEZ (Genscript).

The non-phosphorylatable point mutant AAV-FLEX-FLIM-AKAR^T391A^ was made by site-directed mutagenesis of threonine to alanine at amino acid 391 of the construct AAV-FLEX-FLIM-AKAR.

For the construction of AAV-FLEX-PKIalpha-IRES-mRuby2, the coding region of mouse PKIalpha (GenBank ID: NM_008862) was made by gene synthesis, followed by subcloning of the synthesized fragment into AAV-FLEX-tmeGFP-IRES-nls-mRuby2 via AvrII and BglII (Genscript). AAV-FLEX-tmeGFP-IRES-nls-mRuby2 was constructed by gene synthesis of IRES-nls-mRuby2 (Lam et al., 2012) and subsequent cloning into AAV-FLEX-FLIM-AKAR via XbaI and XhoI (Genscript).

pBS-β-actin Cre was a gift from Susan Dymecki (Harvard Medical School).

### Cell culture and transfection

HEK293T cells were cultured in DMEM (Invitrogen) supplemented with 10% FBS (Invitrogen) at 37°C in 5% CO_2_. They were plated on coverslips in 24-well plates and transfected with plasmids using Lipofectamine 2000 (Invitrogen). Approximately 15–48 h after transfection, the cells were imaged in solutions containing either HEPES-based buffer (containing in mM: 130 KCl, 1 EGTA, 1 MgCl_2_, 25 HEPES, 10 glucose, 20 sucrose, pH with KOH to 7.5 or as specified in the manuscript), or ACSF (containing in mM: 127 NaCl, 2.5 KCl, 25 NaHCO_3_, 1.25 NaH_2_PO_4_, 2 CaCl_2_, 1 MgCl_2_, and 25 glucose) with carbogen (95% O_2_, 5% CO_2_).

For data in Figure 5, pBS-β-actin Cre was cotransfected with AAV-FLEX-FLIM-AKAR, AAV-FLEX-FLIM-AKAR^T391A^ or AAV-FLEX-PKIalpha-IRES-mRuby2.

### Animal husbandry

All procedures for animal husbandry and surgery were performed following protocols approved by the Harvard Standing Committee on Animal Care and in accordance with National Institutes of Health guidelines.

### Brain slice preparations

Organotypic hippocampal slices were cultured from 6 to 8 day old Spraque Dawley rats (Stoppini et al., 1991). The brain was dissected and immediately placed in cold dissection media. Transverse hippocampal slices were cut with 400 μm thickness and placed above a sterile culture insert (Millicell-CM, Millipore) in 6-well plates containing prewarmed culture media. DNA plasmids were biolistically transfected with a Helios Gene Gun (Biorad) 2 days after culturing. Bullets were made with 60 μg of DNA.

For acute slices, mice were anesthetized with isoflurane. For hippocampal slices, the brain of C57BL/6 mice was rapidly dissected out. Horizontal sections were cut at 300 μm thickness using a Leica VT1000S vibratome (Leica Instruments, Nussloch, Germany) in cold sucrose cutting solution containing (in mM) 87 NaCl, 25 NaHCO3, 1.25 NaH2PO4 2.5 KCl, 75 sucrose, 25 glucose, 7.5 MgCl2. For striatal slices, mice first underwent intracardiac perfusion with cold ACSF. Coronal or parasagittal sections were then cut at 300 μm thickness in cold choline cutting solution containing (in mM) 25 NaHCO_3_, 1.25 NaH_2_PO_4_, 2.5 KCl, 7 MgCl_2_, 25 glucose, 1 CaCl2, 110 choline chloride, 11.6 ascorbic acid, and 3.1 pyruvic acid. Slices were transferred to ACSF after sectioning. The slices were incubated at 34°C for 10–15 min and then kept in ACSF at room temperature. Slices were then transferred to a microscope chamber and imaging was performed in perfusing ACSF with a flow rate of 2–4 ml/min. Both cutting and ACSF solutions were constantly bubbled with carbogen.

### *In utero* electroporation

To target hippocampal pyramidal neurons by *in utero* electroporation, glass injection micropipettes were pulled, and the tip broken to be approximately 60 μm in diameter, and beveled at 18° (NARISHIGE, Japan). E15 timed-pregnant female C57BL/6 mice (Charles River, MA, United States) were anesthetized with 2% isoflurane. 1–2 μl of DNA (1 μg/μl) with the dye FastGreen (0.005%) were injected into the left lateral ventricle. The embryo head was then held with a tweezer with round plate electrodes (0.5 mm diameter) and electric pulses were delivered five times per second (50 V, 50 ms) with the cathode placed at the right cortex and the anode at the left cortex (CUY21 electroporator, NEPA GENE, Japan). Warm PBS was dripped onto embryos periodically. The uterus was placed back into the pregnant mother, and the muscle and the skin were sutured separately. Pups were housed with the mother until they were used.

### Virus production and stereotaxic viral injections

AAV-FLEX-FLIM-AKAR was packaged as serotype 8 at University of North Carolina Gene Therapy Center Virus Core Facility. For stereotaxic viral injections, P2–4 pups were anesthetized with isofluorane and placed on a small stereotaxic frame (David Kopf Instruments). For striatal injections, 1 μl of virus (2 × 10^∧^12 genome copies/ml of AAV-FLEX-FLIM-AKAR) were injected into the right hemisphere of either an *Adora2a* BAC-Cre pup (GENSAT, founder line KG139) to target iSPNs (Heintz, 2004; Durieux et al., 2009), or a *Drd1* BAC-Cre pup (GENSAT, founder line EY262) to target dSPNs (Heintz, 2004; Gong et al., 2007). Injection coordinates were approximately lateral 1.5 mm from Bregma, and 2.3 mm beneath the skull. The injection was at a rate of 200 nl/min through a UMP3 microsyringe pump (World Precision Instruments). After injection, pups were returned to their home cage with their mother, and kept for 16–22 days before being used for experimentation.

### Two-photon imaging and fluorescence lifetime imaging microscopy

Two-photon imaging was achieved by a custom-built microscope (Carter and Sabatini, 2004) together with a mode-locked Ti:sappire laser (Chameleon Vision II, 80MHz, Coherent, Santa Clara, CA). Photons were collected with fast photomultiplier tubes (PMTs) (H7422-40MOD Hamamatsu). Excitation wavelengths of 820, 860, and 920 nm were used to excite donor fluorophores of CFP, Turquoise, and meGFP respectively. On the emission path, a 700SP filter (Semrock) was used to filter off excitation light. For meGFP imaging, a 565LP dichroic mirror (Chroma) and 525/50 emission filter (Semrock) were used. For CFP and Turquoise imaging, a 520LP dichroic mirror (Semrock) and 480/40 emission filter (Chroma) were used.

The custom-written software ScanImage (Pologruto et al., 2003) was run in Matlab to acquire imaging data. The FLIM data acquisition and analysis modules were modified from the software from Ryohei Yasuda (Max Planck Florida Institute). Scan mirror control and fluorescence signal acquisition were achieved with the data acquisition (DAQ) board PCI 6110E (National Instruments). An additional DAQ board PCI 6713 (National Instruments) is used to generate frame and line clocks to synchronize ScanImage and the FLIM board SPC-150 (Becker and Hickl GmbH).

The epifluorescence PMTs were used for 2pFLIM, and transfluorescence PMTs for regular imaging. Time-domain single photon counting was used for FLIM and the data were collected in either 64 or 256 time channels. Lifetime decay curve was constructed by comparing times of laser pulses detected by photodiode and photon pulses from the fast PMT.

### Fluorescence lifetime curve fitting and image analysis

Instrument response curve (IRF) for photon spreading was measured with double harmonic generation of urea crystals. It was then used to deconvolve the fluorescence decay curve.

FLIM data were processed by first determining the offset arrival time from a full field-of-view to increase the accuracy of fitting, followed by calculation of FRET fractions of photons for individual regions of interests (ROIs). The processing procedure was as described in Yasuda et al. (2006), Harvey et al. (2008), except that a measured IRF rather than a Gaussian IRF was used to fit the fluorescence decay curve.

### Fluorescence recovery after photobleaching (FRAP)

For FRAP experiments with FLIM-AKAR, 920 nm laser was used to photobleach meGFP. Fluorescence data were collected 1 s before bleaching and then 10 s after a step perturbation of bleaching. Laser pulse width (4, 50, or 100 ms) and laser power were adjusted to give 30–50% bleaching. Three to ten acquisitions were collected for each spine or dendritic region. For FRAP in spines, mushroom spines were selected; for FRAP in dendrites, relatively aspiny regions of dendrites (aspiny in a 5.5 ×5.5 μm square) were selected. Less than 3 spines or dendrites were imaged from each cell. Linescan was used to achieve good temporal resolution of FRAP data.

For processing of FRAP data, fluorescence data from each acquisition was normalized against baseline before photobleaching. The data were then averaged and fitted with a single exponential decay curve (not specifying return to baseline) to calculate τ.

### Statistics

Student *t*-tests (unpaired, assuming unequal variance) were used to compare different conditions. In cases where various reporters were compared with AKAR3.4, Bonferroni correction was used to counteract the problem of multiple comparisons.

### 2-photon photolysis of caged glutamate

A second laser was tuned to 720 nm for 2-photon photolysis of caged glutamate. The bath solution consists of 9 ml of circulating ACSF, with no magnesium, 4 mM CaCl2, 1 μM TTX, 10 μM D-serine, 200 μM Trolox (Sigma), 2 mM pyruvate, 5 U/ml glutamic-pyruvate transaminase (Sigma), and 3.3 mM MNI-glutamate (Tocris). Light power of 75 mW at the back aperture of the 60X (NA1.1) objective was used. Light pulses were delivered at 0.5 ms duration for each pulse, and 40 pulses were delivered in 1 min. 2-photon uncaging was performed adjacent to a spine, and both the spine morphology and FLIM-AKAR response were monitored.

### Pharmacology

Drugs were applied *via* bath perfusion, with the final concentrations in the brackets: forskolin (50 μM), H89 (10 μM), isoproterenol (1 μM), CGS21680 (1 μM), and SKF81297 (1 μM) were from Tocris Bioscience; nigericin (5 μM) was from Sigma. The specified concentration of chemicals were either spiked into the circulating buffer, or premade buffers with the correct drug concentrations were switched from one to another via a custom-made solution exchanger.

## Author note

During the preparation of this manuscript, a single-fluorophore PKA biosensor has been developed that is also 2pFLIM compatible (Bonnot et al., 2014), and future experiments will be needed to compare the detailed characteristics of our sensor and the single-fluorophore PKA biosensor.

## Author contributions

Yao Chen and Bernardo L. Sabatini designed the experiments. Yao Chen, Gary Yellen, and Bernardo L. Sabatini implemented the hardware and software of the FLIM setup. Jessica L. Saulnier generated organotypic slices and performed some *in utero* electroporation surgeries. Yao Chen performed the rest of the experiments and data analysis. Yao Chen, Gary Yellen, and Bernardo L. Sabatini wrote the manuscript.

### Conflict of interest statement

The authors declare that the research was conducted in the absence of any commercial or financial relationships that could be construed as a potential conflict of interest.

## Supplementary material

Supplementary Figure 1 **Diagram illustrating how FRET changes fluorescence lifetime**. Free donor fluorophores show a single exponential decay of fluorescence lifetime distribution (green). When donor and acceptor fluorophores interact via FRET, an additional decay process occurs, resulting in bi-exponential decay and shorter lifetime (red). P_free_ and P_FRET_ represent the fractions of donors that are free and that have undergone FRET respectively. P_free_ + P_FRET_ = 1.

Supplementary Table 1 **Optical characteristics of the three donors and two acceptors used in the 4 PKA reporters**. The brightness is relative to eGFP. The optical characteristics are based on literature (Piston et al.; Ganesan et al., 2006; Murakoshi et al., 2008) and our own data.
