# Corrigendum: A PKA activity sensor for quantitative analysis of endogenous GPCR signaling *via* 2-photon FRET-FLIM imaging

**DOI:** 10.3389/fphar.2016.00046

**Published:** 2016-02-25

**Authors:** Yao Chen, Jessica L. Saulnier, Gary Yellen, Bernardo L. Sabatini

**Affiliations:** ^1^Howard Hughes Medical InstituteBoston, MA, USA; ^2^Department of Neurobiology, Harvard Medical SchoolBoston, MA, USA

**Keywords:** PKA, FLIM, neuromodulation, cAMP, FLIM-AKAR, GPCR, glutamate, dendritic spine

Reason for Corrigendum:

There was an error in the Materials and Methods section, subsection *In utero* Electroporation of our Original Research article “A PKA activity sensor for quantitative analysis of endogenous GPCR signaling *via* 2-photon FRET-FLIM imaging.” The round plate electrodes were 5 mm in diameter, instead of 0.5 mm as stated before.

The authors apologize for the mistake.

This error does not change the scientific conclusions of the article in any way.

## Author contributions

YC wrote the Corrigendum. All authors agree to the request for changes.

### Conflict of interest statement

The authors declare that the research was conducted in the absence of any commercial or financial relationships that could be construed as a potential conflict of interest.

