# Supplementary figures and images for: A PKA activity sensor for quantitative analysis of endogenous GPCR signaling via 2-photon FRET-FLIM imaging
Source: Front Pharmacol. 2014 Apr 2;5:56. doi: 10.3389/fphar.2014.00056 (PMC3980114; doi:10.3389/fphar.2014.00056)

Supplementary Figure 1

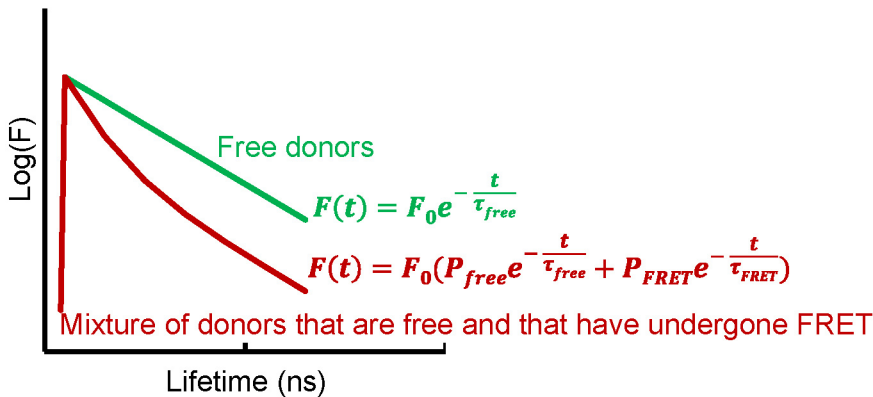

Supplement: Supplementary Figure 1 — Diagram illustrating how FRET changes fluorescence lifetime. Free donor fluorophores show a single exponential decay of fluorescence lifetime distribution (green). When donor and acceptor fluorophores interact via FRET, an additional decay process occurs, resulting in bi-exponential decay and shorter lifetime (red). Pfree and PFRET represent the fractions of donors that are free and that have undergone FRET respectively. Pfree + PFRET = 1. [file Presentation1.PDF]
