# Supplementary material for: A PKA activity sensor for quantitative analysis of endogenous GPCR signaling via 2-photon FRET-FLIM imaging
Source: Front Pharmacol. 2014 Apr 2;5:56. doi: 10.3389/fphar.2014.00056 (PMC3980114; doi:10.3389/fphar.2014.00056)
Supplement: Supplementary Table 1 — Optical characteristics of the three donors and two acceptors used in the 4 PKA reporters. The brightness is relative to eGFP. The optical characteristics are based on literature (Piston et al.; Ganesan et al., 2006; Murakoshi et al., 2008) and our own data. [file DataSheet1.DOCX]

**Supplementary Table 1: Optical characteristics of the three donors and two acceptors used in the 4 PKA reporters.** The brightness is relative to eGFP. The optical characteristics are based on literature ([Piston et al.](#_ENREF_42);[Ganesan et al., 2006](#_ENREF_22);[Murakoshi et al., 2008](#_ENREF_39)) and our own data.

| **Supplementary Table 1** | |  |  |
| --- | --- | --- | --- |
|  |  |  |  |
|  | **Donor** | | |
| **Characteristics** | **CFP** | **Turquoise** | **eGFP** |
| **Relative brightness** | 39 | 85 | 100 |
| **Lifetime rundown** | High | Medium | Low |
| **Fluorescence decay** | Multi-exponential | Mono-exponential | Mono-exponential |
|  |  |  |  |
|  | **Acceptor** | |  |
| **Characteristics** | **Venus** | **sREACH** |  |
| **Relative brightness** | 156 | 1 |  |
